# Plasticity Response in the Contralesional Hemisphere after Subtle Neurotrauma: Gene Expression Profiling after Partial Deafferentation of the Hippocampus

**DOI:** 10.1371/journal.pone.0070699

**Published:** 2013-07-25

**Authors:** Daniel Andersson, Ulrika Wilhelmsson, Michael Nilsson, Mikael Kubista, Anders Ståhlberg, Marcela Pekna, Milos Pekny

**Affiliations:** 1 Center for Brain Repair and Rehabilitation, Department of Clinical Neuroscience and Rehabilitation, Institute of Neuroscience and Physiology, Sahlgrenska Academy at University of Gothenburg, Gothenburg, Sweden; 2 Hunter Medical Research Institute, Newcastle, Australia; 3 Institute of Biotechnology, Academy of Sciences of the Czech Republic, Prague, Czech Republic; and TATAA Biocenter, Gothenburg, Sweden; 4 Department of Pathology, Institute of Biomedicine, Sahlgrenska Academy at University of Gothenburg, Gothenburg, Sweden; Univ. Kentucky, United States of America

## Abstract

Neurotrauma or focal brain ischemia are known to trigger molecular and structural responses in the uninjured hemisphere. These responses may have implications for tissue repair processes as well as for the recovery of function. To determine whether the plasticity response in the uninjured hemisphere occurs even after a subtle trauma, we subjected mice to a partial unilateral deafferentation of the hippocampus induced by stereotactically performed entorhinal cortex lesion (ECL). The expression of selected genes was assessed by quantitative real-time PCR in the hippocampal tissue at the injured side and the contralesional side at day 4 and 14 after injury. We observed that expression of genes coding for synaptotagmin 1, ezrin, thrombospondin 4, and C1q proteins, that have all been implicated in the synapse formation, re-arrangement and plasticity, were upregulated both in the injured and the contralesional hippocampus, implying a plasticity response in the uninjured hemisphere. Several of the genes, the expression of which was altered in response to ECL, are known to be expressed in astrocytes. To test whether astrocyte activation plays a role in the observed plasticity response to ECL, we took advantage of mice deficient in two intermediate filament (nanofilament) proteins glial fibrillary acidic protein (GFAP) and vimentin (*GFAP^−/−^Vim^−/−^*) and exhibiting attenuated astrocyte activation and reactive gliosis. The absence of GFAP and vimentin reduced the ECL-induced upregulation of thrombospondin 4, indicating that this response to ECL depends on astrocyte activation and reactive gliosis. We conclude that even a very limited focal neurotrauma triggers a distinct response at the contralesional side, which at least to some extent depends on astrocyte activation.

## Introduction

Experience-dependent adaptation and learning are at the structural level based on dendritic and axonal arborization, spine density, synapse number and size, receptor density and in some brain regions also the generation of new neurons. Together, these structural constituents of neural plasticity also contribute to recovery of function after CNS injury [1].

In recent years it has become increasingly apparent that the contralateral hemisphere plays an important role in the recovery of function after brain injury [1]. Indeed, several studies show that the gene expression profiles in the contralesional hemisphere are altered both early (hours) [2] and late (days) [3] after experimental ischemic stroke and could play a role in functional recovery [3], [4]. However, whether subtle indirect injury to the brain elicits any detectable contralesional changes in gene expression, in particular the expression of genes involved in neural plasticity, is unknown.

Astrocytes, the most common cells of the mammalian brain, respond to all forms of injuries and diseases of the brain by molecular, cellular and functional changes in a process known as reactive gliosis or astrogliosis [5], [6], [7], [8]. Astrocytes also play an important role in the regulation of several constituents of neural plasticity including cell genesis, control of the number of neuronal synapses and synapse function [1], [9]. Astrocyte-derived factors participate in synaptic plasticity by inducing synapse formation and maturation [10], [11], [12]. By inducing the expression of complement component C1q in neurons, astrocytes play a role in the elimination of supernumerary synapses during development and as an initial step in neurodegeneration [13]. Astrocytes are also involved in regulating the number of synapses after injury [14].

In this study we sought to determine the gene expression profile of selected genes known to be involved in neural plasticity in the affected and contralesional hippocampus at 4 and 14 days following stereotactically performed unilateral entorhinal cortex lesion (ECL). In this injury model, hippocampus is not directly injured but is indirectly affected via partial deafferentation and Wallerian degeneration [15], [16]. To elucidate the role of activated astrocytes in the contralesional response to ECL, we used mice with genetically ablated ability to express glial fibrillary acidic protein (GFAP) and vimentin, two intermediate filament (nanofilament) proteins, the upregulation of which is a hallmark of astrocyte activation in response to CNS injury [7], [14], [17], [18].

## Materials and Methods

### Ethic statement

All experiments were approved by the Gothenburg Committee on the Ethics of Animal Experiments (permit numbers 29-2006 and 57-2009) and performed in agreement with the guidelines on research animal welfare (L150) from the Swedish National Board of Agriculture.

### Surgical procedures

Unilateral entorhinal cortex lesioning was performed as described [19], [20] in 6–15 months old age matched wild-type and *GFAP^−/−^Vim^−/−^* males (4 to 7 mice per group and time point) of C57Bl/6-129Ola-129Sv mixed genetic background maintained in a barrier animal facility. Mice were deeply anesthetized through intraperitoneal injection of 2.5% avertin, placed in a stereotactic frame, and a hole was drilled through the skull. A retractable wire knife (David Kopf Instruments, Tujunga, CA) was lowered 1 mm down from the dura +3.6 mm laterally and −0.1 mm posterior to lambda. The wire knife was extended 2 mm horizontally and then lowered 2 mm twice at +30° and −135° to avoid the hippocampal formation. The mice were kept in heated cages until they recovered from anesthesia.

### RNA extraction

Mice were killed by cervical dislocation, brains were removed and hippocampus dissected out in ice-cold PBS. Samples were after dissection immediately immersed in RNA*later* (Qiagen, Hilden, Germany) before storage at −80C. Homogenization was performed by adding 1 ml TRizol Reagent (Life Technologies, Carlsbad, CA, USA), one 5 mm steal bead (Qiagen) to each sample before shaking for 2×5 mins at 25 Hz using Tissuelyser (Qiagen). Following homogenization total RNA was extracted by using the protocol for TRIzol Reagent according to manufacturer's instructions, followed by DNase digestion of RNA prior to purification by RNeasy Mini Kit (both Qiagen). RNA concentration was determined by using a NanoDrop 1000 Spectrophotometer (Thermo Scientific, Wilmington, DE, USA) and RNA integrity was checked using the Agilent 2100 Bioanalyzer (Agilent Technologies, Santa Clara, CA, USA).

### Reverse Transcription and Quantitative Real-Time PCR

cDNA was generated using iScript cDNA Synthesis Kit (Bio-Rad Laboratories, Hercules, Ca, USA) with a mixture of random primers and oligo(dT) primers, according to the manufacturer's instructions. The reverse transcription was run in duplicate in 24 µl reactions [21] using 1.2 µg of total RNA per cDNA reaction. Following cDNA synthesis, the samples were diluted with nuclease-free water to a concentration corresponding to 10 ng/µl of total RNA. SYBR-Green I-based PCR assays for individual genes were designed using Primer3 (http://frodo.wi.mit.edu/) and NetPrimer (Premier Biosoft, Palo Alto, CA, USA). Formation of expected PCR product was confirmed by 1.5% agarose gel electrophoresis and melting curve analysis for all assays. The assay specific PCR efficiencies were determined based on dilution series of the PCR product [22]. Real-time PCR experiments were performed on a Rotor-Gene 3000 or 6000 (Corbett Research, Sydney, Australia). Temperature profile of real-time PCR was 95°C for 3 minutes, followed by 40 cycles at 95°C for 20 seconds, 60°C for 20 seconds and 72°C for 20 seconds. Total reaction volume of real-time PCR experiments was 20 µl, containing iQ SYBR Green Supermix (Bio-Rad) and 0.4 µM of forward and reverse primers, respectively (Eurofins MWG Operon, Ebersberg, Germany) and 2 µl cDNA template. Reference genes were evaluated using the Reference Gene Panel Mouse (TATAA Biocenter, Gothenburg, Sweden) and NormFinder [23] and TATAA-box binding protein (*TBP*) and total RNA were used to normalize the mRNA expression levels. Data were analyzed as previously described [22]. For further PCR assay information, see Table 1.

**Table 1:** PCR assay information and primer sequences.

Gene symbol	Full name	NCBI accession number	Intron spanning	PCR efficiency (%)	Forward primer (5′-3′) and reverse primer (5′-3′)
*GFAP*	Glial fibrillary acidic protein	NM_010277.3	Yes	97	AACCGCATCACCATTCCT CGCATCTCCACAGTCTTTACC
*Vim*	Vimentin	NM_011701.4	Yes	89	CAGATGCGTGAGATGGAAGA GTTGGCAGAGGCAGAGAAA
*Ezr*	Ezrin	NM_009510.2	Yes	90	GGCACCTGACTTTGTGTTCT CGTCTCTCGCCTCTTCTTC
*Thbs4*	Thrombospondin 4	NM_011582.3	Yes	86	CGACTTGGTGTGTTCTGCTT CAGTTGTGGGATTGCTTCTTG
*Syt1*	Synaptotagmin 1	NM_001252341.1	Yes	95	GGATGTGGGTGGCTTATCTG CCAATCTTGTCATAGTCCAAAAC
*Gap43*	Growth associated protein 43	NM_008083.2	Yes	73	CACTGATAACTCCCCGTCCT GGTCTTCTTTACCCTCATCCTGT
*Syp*	Synaptophysin	NM_009305.2	Yes	86	CCACCTCCTTCTCCAATCAG ACAGCAAAGACAGGGTCTCC
*C1qc*	Complement component 1, q subcomponent, C chain	NM_007574.2	Yes	97	CCTGCTGCTGCTGTTTCTT GATTCCTGGCTCTCCCTTG
*C3*	Complement component 3	NM_009778.2	Yes	78	GCCTCTCCTCTGACCTCTGG AGTTCTTCGCACTGTTTCTGG
*TBP*	TATAA-box binding protein	Not available	Yes	89	Mouse Endogenous Control Gene Panel, TATAA Biocenter AB, Gothenburg, Sweden

### Statistics

Data were analyzed using PASW Statistics 18.0.0 (IBM Corporation). One-way ANOVA was used followed by post hoc analysis (Bonferroni correction). Differences were considered significant at p<0.05. Values are presented as mean ± SEM.

## Results

### GFAP and vimentin are upregulated after partial hippocampal deafferentation

**Figure 1:**
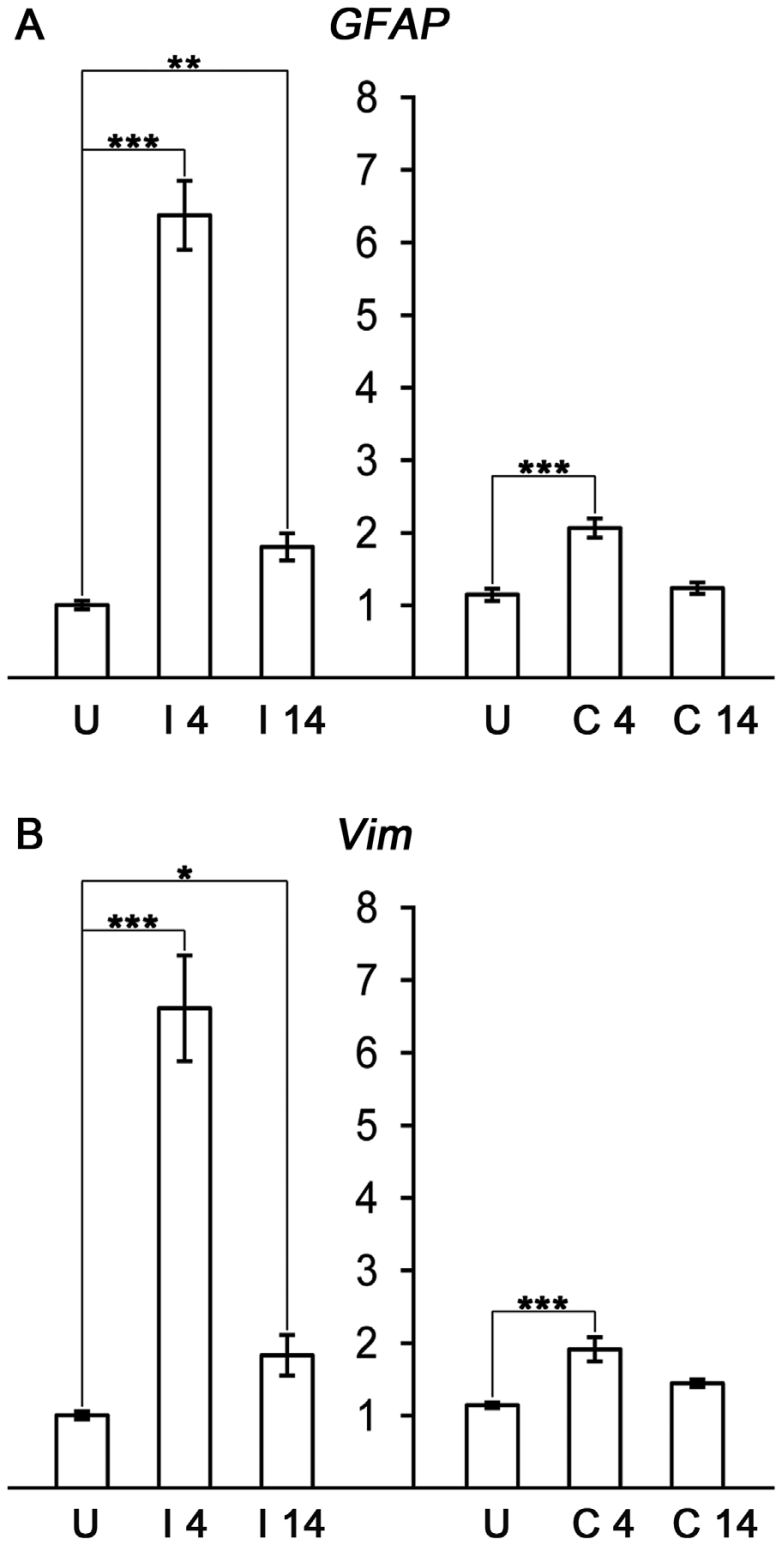
*GFAP* and *Vim* upregulation after ECL at the injured and contralesional side. (A) *GFAP* and (B) vimentin (*Vim*) mRNA is upregulated in the hippocampal tissue at the injured side (I) at both 4 and 14 days after injury and at the contralesional side (C) at day 4. U, uninjured control; * p<0.05; ** p<0.01; *** p<0.001.

To confirm that ECL resulted in the expected indirect injury and astrocyte response in the hippocampus on the injured side, we first determined the mRNA levels of GFAP and vimentin (*Vim*), the increased expression of which is a hallmark of astrocyte activation. We observed that both at 4 and 14 days after ECL, GFAP and vimentin expression was significantly increased in the deafferented hippocampus (*GFAP* by 534% and 79%, respectively, and *Vim* by 558% and 82%, respectively). Moreover, both genes were upregulated also in the contralesional hippocampus 4 days after ECL (by 80% and 68% for *GFAP* and *Vim*, respectively; Fig. 1) suggesting that also astrocytes in the unlesioned hippocampus respond to the insult.

### Ezrin, synaptotagmin 1 and thrombospondin 4 are upregulated in the deafferented hippocampus

**Figure 2:**
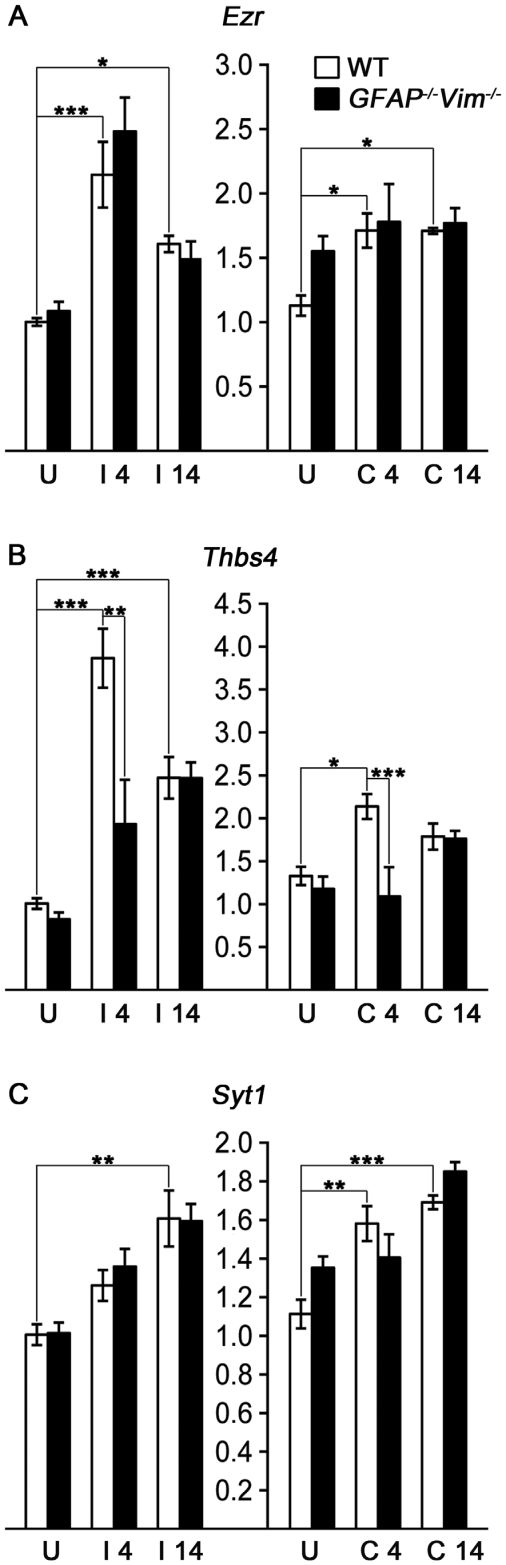
*Ezr*, *Thbs4* and *Syt1* upregulation after ECL at the injured and contralesional side. (A) Ezrin (*Ezr*) and (B) thrombospondin 4 (*Thbs4*) mRNA is upregulated in hippocampal tissue at the injured (I) side at both 4 and 14 days after injury. (C) Synaptotagmin 1 (*Syt1*) mRNA is upregulated in the injured side at day 14. At the contralesional side (C) at day 4, all three genes show mRNA upregulation. *Ezr* and *Syt1* also show mRNA upregulation on the contralesional side at day 14. *Thbs4* mRNA levels in the hippocampal tissue on both the injured and contralesional side are lower in *GFAP^−/−^Vim^−/−^* mice compared to wild-type mice at 4 days after ECL. U, uninjured control; * p<0.05; ** p<0.01; *** p<0.001.

To assess the response of synaptic plasticity markers, we measured the mRNA levels of ezrin (*Ezr*), thrombospondin 4 (*Thbs4*) and synaptotagmin (*Syt1*) that all participate in synaptic remodeling. Specifically, ezrin has been shown to be involved in the structural plasticity of processes of perisynaptic astrocytes [24]. Thrombospondin 4 is expressed and localized to synapses and neuromuscular junction at adulthood [25] and stimulates neurite outgrowth [25], [26]. Synaptotagmin is upregulated and aggregates at the presynaptic sites during synapse formation between adult neurons [27]. *Ezr* and *Thbs4* mRNA was significantly upregulated in the deafferented hippocampus both 4 and 14 days after ECL (*Ezr* by 114% and 60%, respectively, and *Thbs4* by 284% and 145%, respectively), whereas *Syt1* was upregulated only at 14 days after ECL (by 60%). *Ezr* and *Syt1* were also upregulated in the contralesional hippocampus at both time points (*ezrin* by 52% and 51%, respectively, and *Syt1* by 42% and 52%, respectively). *Thbs4* mRNA was increased in the contralesional hippocampus only 4 days after ECL (by 61%; Fig. 2).

**Figure 3:**
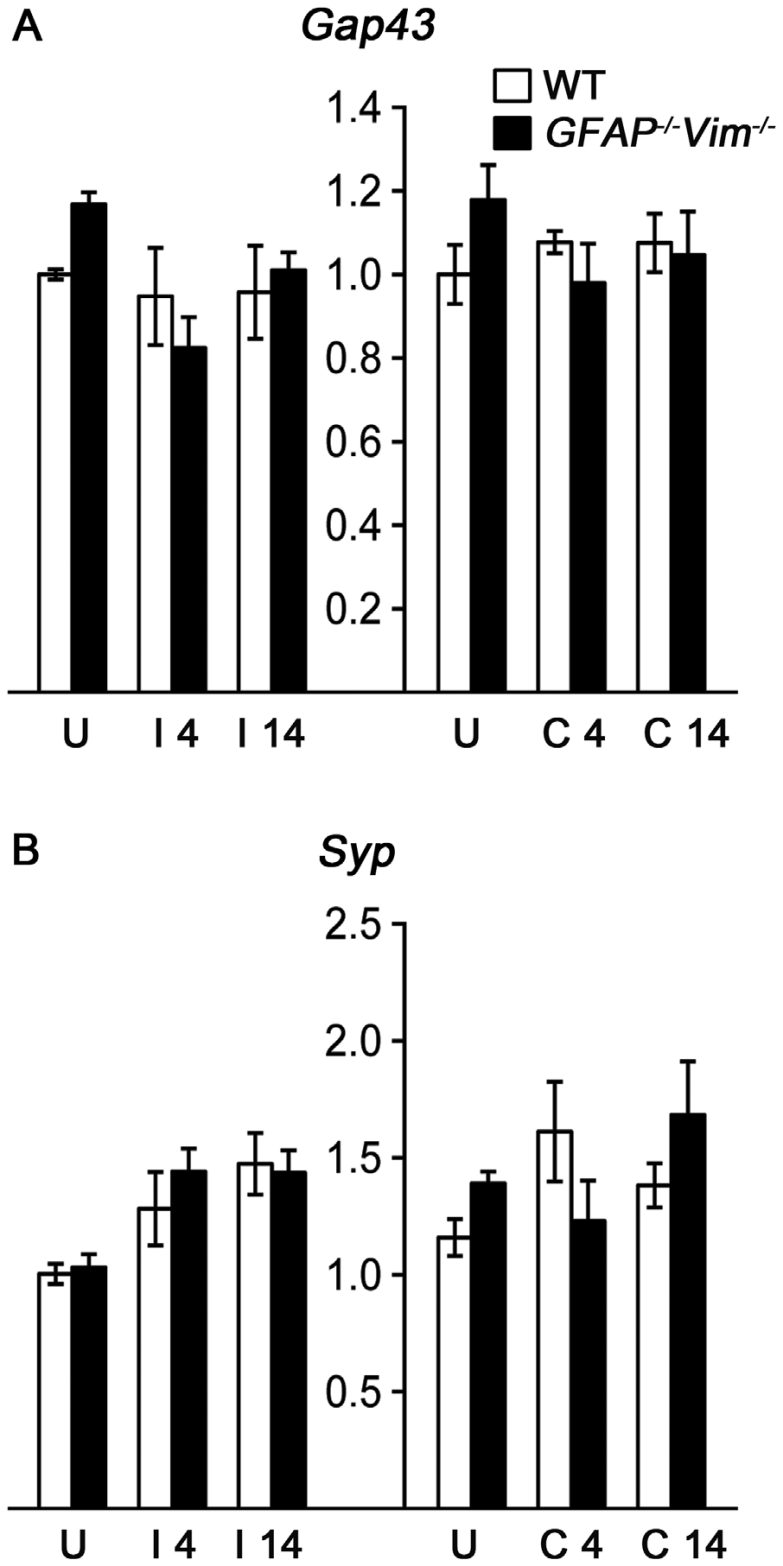
*Gap43* and *Syp* expression are unaffected by ECL. (A) Growth associated protein 43 (*Gap43*) and (B) synaptophysin (*Syp*) mRNA levels in hippocampal tissue remained unchanged at 4 and 14 days after injury. U, uninjured control; I, injured hippocampus; C, contralesional hippocampus.

In contrast, we did not observe any changes in the mRNA expression of a marker of neurite outgrowth, growth associated protein 43 (*Gap43*) [28], [29] that is also induced in reactive astrocytes [30](data not shown), or synaptophysin (*Syp*), a protein associated with activity-dependent competitive synapse-formation [31] (Fig. 3).

To assess whether the expression of the above genes depends on astrocyte activation and reactive gliosis, we compared the gene expression in wild-type and *GFAP^−/−^Vim^−/−^* mice that show attenuated astrocyte activation and reactive gliosis [17], [18], [32], [33], [34]. We found that 4 days after ECL, the expression of *Thbs4* was significantly lower in both the affected and contralesional hippocampus in *GFAP^−/−^Vim^−/−^* compared to wild-type mice (by 50% and 49%, respectively; Fig. 2).

### Complement components C1q and C3 are upregulated following ECL

**Figure 4:**
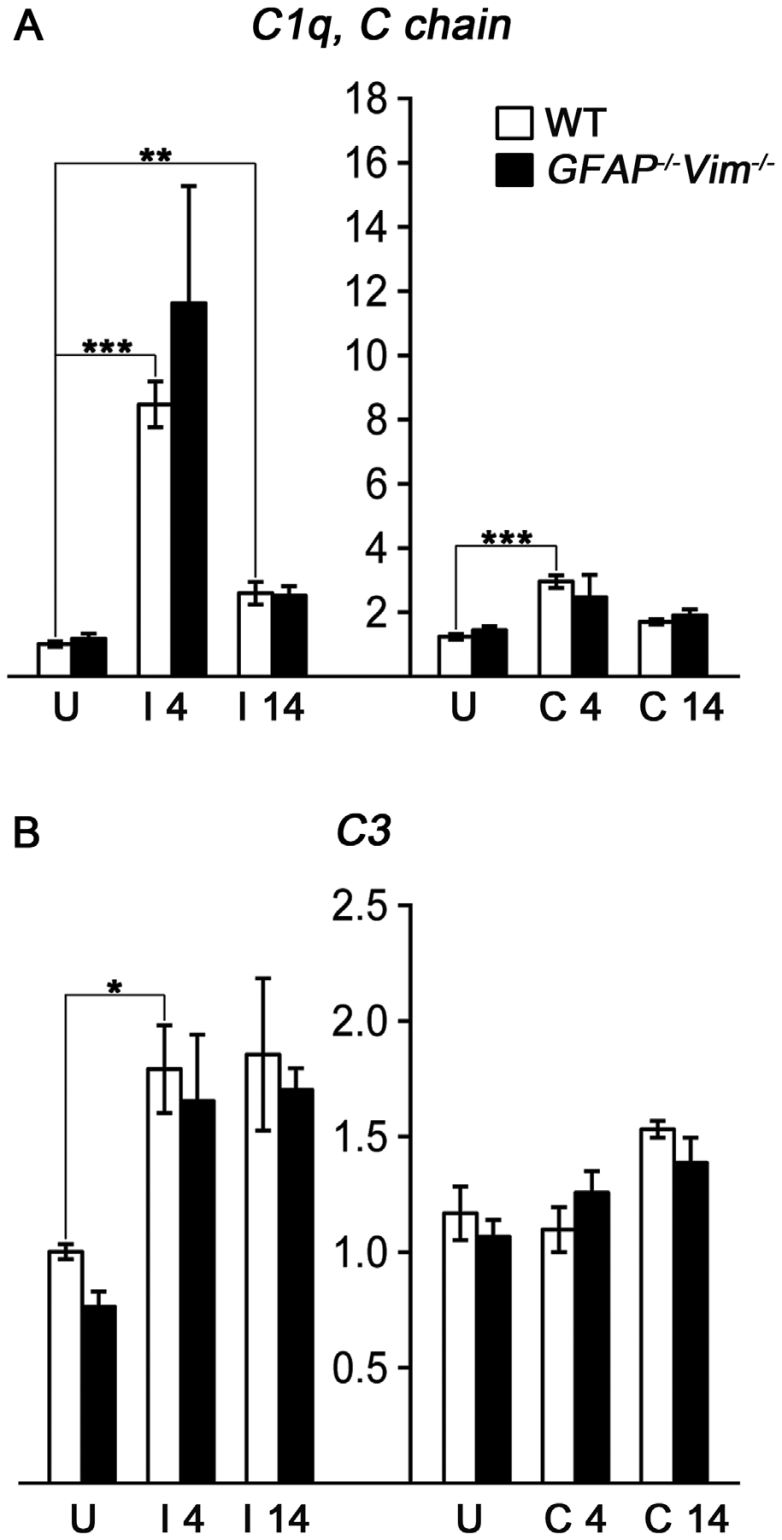
Complement components C1q and C3 upregulation following ECL. (A) Complement component C1q (here represented by *C1q, C chain*) mRNA is upregulated in the hippocampal tissue at the injured side (I) at both 4 and 14 days after injury and (B) *C3* mRNA is upregulated at 4 days after injury. *C1q* is also upregulated at the contralesional side (C) at day 4. U, uninjured control; * p<0.05; ** p<0.01; *** p<0.001.

As the complement system has been shown to be involved in synaptic elimination both during development and in pathological situations such as glaucoma [13] or sciatic nerve injury [35], we determined the expression levels of the complement components C1q and C3. At 4 days after ECL, both *C1q* (here represented by complement component 1, q subcomponent, C chain; *C1qc*) and *C3* mRNA was significantly upregulated in the affected hippocampus (by 735% and 79%, respectively). *C1qc* mRNA was still increased at 14 days after injury (by 156%). In addition, *C1qc* but not *C3* mRNA was significantly increased in the contralesional hippocampus 4 days after lesion (by 138%; Fig. 4).

The expression pattern of *C1qc* and *C3* mRNA was not altered in *GFAP^−/−^Vim^−/−^* as compared to wild-type mice suggesting that the expression of these genes is independent of astrocyte activation or more specifically, the presence of GFAP and vimentin.

## Discussion

Ischemic stroke leads to changes in the expression of a large number of genes both in the ischemic as well as in the contralesional hemisphere [2], [3]. There is a growing body of evidence showing that the contralateral hemisphere plays a role in the recovery of function after brain injury [1] and changes in gene expression profiles in brain regions contralateral to the ischemia have been implicated in functional recovery [3], [4]. Here we report that a partial deafferentation of the hippocampus leads to upregulation of GFAP and vimentin mRNA in the affected as well as contralesional hippocampal tissue. These findings demonstrate that even a very mild focal injury in the CNS induces glial cell activation also in the contralateral hemisphere. Further, this glial cell response is less pronounced on the contralesional side but has the same temporal pattern in both hemispheres. Whether these changes are mediated via interhippocampal neuronal connections, signals conveyed through the astrocyte syncytium or other mechanisms remains to be experimentally addressed.

We further demonstrate that several genes involved in synaptic re-organization and plasticity, namely thrombospondin 4, ezrin and synaptotagmin 1 are upregulated both in the affected and contralesional hippocampus. Remarkably, the expression levels of ezrin and synaptotagmin 1 in the two hemispheres were comparable pointing to similar degree of synaptic plasticity in the affected and contralesional hippocampus. Although ezrin is involved in the structural plasticity of processes of perisynaptic astrocytes [24], we did not observe any effect of the absence of GFAP and vimentin on the *Ezr* mRNA expression profiles prior to or after ECL. These results indicate that GFAP and vimentin are not necessary for the regulation of ezrin expression, although the morphology of astrocyte processes in response to ECL is clearly altered in *GFAP^−/−^Vim^−/−^* mice [14]. Thrombospondin 4, localized to synapses and neuromuscular junctions [25], is also involved in the regulation of neurite outgrowth [25], [26]. Thrombospondin 4 is expressed in astrocytes, its expression is upregulated after spinal nerve injury; injury-induced spinal thrombospondin 4 contributes to spinal presynaptic hypersensitivity and neuropathic pain states [36]. Our data show that the expression profile of *Thbs4* was clearly affected by the absence of GFAP and vimentin such as the early (4 days post ECL) upregulation of *Thbs4* expression observed in wild-type mice was abrogated in both hemispheres in *GFAP^−/−^Vim^−/−^* mice. Thus, GFAP and vimentin expression and normal reactive gliosis are necessary for the upregulation of thrombospondin 4 in response to injury in both the affected and contralesional brain tissue. Our findings also point to the intriguing possibility of inhibiting thrombospondin 4-mediated maladaptive neural plasticity through the modulation of reactive gliosis, although the role of thrombospondin 4 in the hippocampus and contralesional plasticity remains to be determined.

Remarkably, we did not observe any changes in the mRNA expression of *Gap43*, a marker of neurite outgrowth [28], [29], that was reported to be upregulated in reactive astrocytes surrounding cerebral infarct as early as 1 day after ischemia induction [30]. Thus, more severe injury or different time point after injury may be necessary for the detection of upregulation of *Gap43* in the hippocampus.

Activation of the complement cascade plays a role in injury induced inflammation and tissue damage. However, complement proteins C1q and C3 have been shown to be involved in the elimination of synapses from the maturing [13] and injured [35] or degenerating neurons [13] and thus participate in synaptic plasticity in the developing as well as adult CNS. In addition, *C3* mRNA and mRNA of genes that code for components of the C1 complex are upregulated in reactive astrocytes after ischemia as well as lipopolysaccharide induced neuroinflammation [30]. *C3* mRNA is also upregulated in sprouting axons 7 days after stroke [37]. Here, we provide evidence that both genes are upregulated in the hippocampal tissue after ECL and *C1qc* mRNA is increased also in the contralesional hippocampus. The role of C1q and C3 in synaptic remodeling of the deafferented hippocampus and the role of complement in the contralesional synaptic plasticity and functional recovery warrant further investigation.

In conclusion, we show that genes associated with astrocyte activation and neural plasticity show very pronounced and prolonged response to even a very mild and indirect injury to the brain tissue, that this response is clearly detectable also in the contralesional hemisphere and the upregulation of some of the plasticity-related genes is dependent on reactive gliosis or the presence of astrocyte intermediate filament proteins GFAP and vimentin.

## References

[1] Pekna M, Pekny M, Nilsson M (2012) Modulation of neural plasticity as a basis for stroke rehabilitation. Stroke 43: 2819–2828. 22923444 10.1161/STROKEAHA.112.654228

[2] Hori M, Nakamachi T, Rakwal R, Shibato J, Nakamura K, et al (2012) Unraveling the ischemic brain transcriptome in a permanent middle cerebral artery occlusion mouse model by DNA microarray analysis. Dis Model Mech 5: 270–283. 22015461 10.1242/dmm.008276 PMC3291648

[3] Buga AM, Sascau M, Pisoschi C, Herndon JG, Kessler C, et al (2008) The genomic response of the ipsilateral and contralateral cortex to stroke in aged rats. J Cell Mol Med 12: 2731–2753. 18266980 10.1111/j.1582-4934.2008.00252.x PMC3828887

[4] Kim MW, Bang MS, Han TR, Ko YJ, Yoon BW, et al (2005) Exercise increased BDNF and trkB in the contralateral hemisphere of the ischemic rat brain. Brain Res 1052: 16–21. 16054599 10.1016/j.brainres.2005.05.070

[5] Eng LF, Ghirnikar RS (1994) GFAP and astrogliosis. Brain Pathol 4: 229–237. 7952264 10.1111/j.1750-3639.1994.tb00838.x

[6] Hernandez MR, Agapova OA, Yang P, Salvador-Silva M, Ricard CS, et al (2002) Differential gene expression in astrocytes from human normal and glaucomatous optic nerve head analyzed by cDNA microarray. Glia 38: 45–64. 11921203 10.1002/glia.10051

[7] Pekny M, Nilsson M (2005) Astrocyte activation and reactive gliosis. Glia 50: 427–434. 15846805 10.1002/glia.20207

[8] Sofroniew MV (2009) Molecular dissection of reactive astrogliosis and glial scar formation. Trends Neurosci 32: 638–647. 19782411 10.1016/j.tins.2009.08.002 PMC2787735

[9] Parpura V, Heneka MT, Montana V, Oliet SH, Schousboe A, et al (2012) Glial cells in (patho)physiology. J Neurochem 121: 4–27. 22251135 10.1111/j.1471-4159.2012.07664.x PMC3304021

[10] Nagler K, Mauch DH, Pfrieger FW (2001) Glia-derived signals induce synapse formation in neurones of the rat central nervous system. J Physiol 533: 665–679. 11410625 10.1111/j.1469-7793.2001.00665.x PMC2278670

[11] Christopherson KS, Ullian EM, Stokes CC, Mullowney CE, Hell JW, et al (2005) Thrombospondins are astrocyte-secreted proteins that promote CNS synaptogenesis. Cell 120: 421–433. 15707899 10.1016/j.cell.2004.12.020

[12] Eroglu C, Allen NJ, Susman MW, O'Rourke NA, Park CY, et al (2009) Gabapentin receptor alpha2delta-1 is a neuronal thrombospondin receptor responsible for excitatory CNS synaptogenesis. Cell 139: 380–392. 19818485 10.1016/j.cell.2009.09.025 PMC2791798

[13] Stevens B, Allen NJ, Vazquez LE, Howell GR, Christopherson KS, et al (2007) The classical complement cascade mediates CNS synapse elimination. Cell 131: 1164–1178. 18083105 10.1016/j.cell.2007.10.036

[14] Wilhelmsson U, Li L, Pekna M, Berthold CH, Blom S, et al (2004) Absence of glial fibrillary acidic protein and vimentin prevents hypertrophy of astrocytic processes and improves post-traumatic regeneration. J Neurosci 24: 5016–5021. 15163694 10.1523/JNEUROSCI.0820-04.2004 PMC6729371

[15] Deller T, Del Turco D, Rappert A, Bechmann I (2007) Structural reorganization of the dentate gyrus following entorhinal denervation: species differences between rat and mouse. Prog Brain Res 163: 501–528. 17765735 10.1016/S0079-6123(07)63027-1

[16] Turner DA, Buhl EH, Hailer NP, Nitsch R (1998) Morphological features of the entorhinal-hippocampal connection. Prog Neurobiol 55: 537–562. 9670217 10.1016/s0301-0082(98)00019-7

[17] Eliasson C, Sahlgren C, Berthold CH, Stakeberg J, Celis JE, et al (1999) Intermediate filament protein partnership in astrocytes. J Biol Chem 274: 23996–24006. 10446168 10.1074/jbc.274.34.23996

[18] Pekny M, Johansson CB, Eliasson C, Stakeberg J, Wallen A, et al (1999) Abnormal reaction to central nervous system injury in mice lacking glial fibrillary acidic protein and vimentin. J Cell Biol 145: 503–514. 10225952 10.1083/jcb.145.3.503 PMC2185074

[19] Pekny M, Porritt MJ, de Pablo Y, Wilhelmsson U (2013) Reactive Astrocytes, Astrocyte Intermediate Filament Proteins, and Their Role in the Disease Pathogenesis In: Dermietzel R, The Cytoskeleton: Imaging, Isolation, and Interaction: Springer protocols. pp 299–319.

[20] Wilhelmsson U, Bushong EA, Price DL, Smarr BL, Phung V, et al (2006) Redefining the concept of reactive astrocytes as cells that remain within their unique domains upon reaction to injury. Proc Natl Acad Sci U S A 103: 17513–17518. 17090684 10.1073/pnas.0602841103 PMC1859960

[21] Stahlberg A, Kubista M, Pfaffl M (2004) Comparison of reverse transcriptases in gene expression analysis. Clin Chem 50: 1678–1680. 15331507 10.1373/clinchem.2004.035469

[22] Stahlberg A, Zoric N, Aman P, Kubista M (2005) Quantitative real-time PCR for cancer detection: the lymphoma case. Expert Rev Mol Diagn 5: 221–230. 15833051 10.1586/14737159.5.2.221

[23] Andersen CL, Jensen JL, Orntoft TF (2004) Normalization of real-time quantitative reverse transcription-PCR data: a model-based variance estimation approach to identify genes suited for normalization, applied to bladder and colon cancer data sets. Cancer Res 64: 5245–5250. 15289330 10.1158/0008-5472.CAN-04-0496

[24] Lavialle M, Aumann G, Anlauf E, Prols F, Arpin M, et al (2011) Structural plasticity of perisynaptic astrocyte processes involves ezrin and metabotropic glutamate receptors. Proc Natl Acad Sci U S A 108: 12915–12919. 21753079 10.1073/pnas.1100957108 PMC3150955

[25] Arber S, Caroni P (1995) Thrombospondin-4, an extracellular matrix protein expressed in the developing and adult nervous system promotes neurite outgrowth. J Cell Biol 131: 1083–1094. 7490284 10.1083/jcb.131.4.1083 PMC2200004

[26] Dunkle ET, Zaucke F, Clegg DO (2007) Thrombospondin-4 and matrix three-dimensionality in axon outgrowth and adhesion in the developing retina. Exp Eye Res 84: 707–717. 17320079 10.1016/j.exer.2006.12.014

[27] Gardzinski P, Lee DW, Fei GH, Hui K, Huang GJ, et al (2007) The role of synaptotagmin I C2A calcium-binding domain in synaptic vesicle clustering during synapse formation. J Physiol 581: 75–90. 17317745 10.1113/jphysiol.2006.127472 PMC2075219

[28] Benowitz LI, Routtenberg A (1997) GAP-43: an intrinsic determinant of neuronal development and plasticity. Trends Neurosci 20: 84–91. 9023877 10.1016/s0166-2236(96)10072-2

[29] Pfenninger KH, de la Houssaye BA, Helmke SM, Quiroga S (1991) Growth-regulated proteins and neuronal plasticity. A commentary. Mol Neurobiol 5: 143–151. 1823138 10.1007/BF02935543

[30] Zamanian JL, Xu L, Foo LC, Nouri N, Zhou L, et al (2012) Genomic analysis of reactive astrogliosis. J Neurosci 32: 6391–6410. 22553043 10.1523/JNEUROSCI.6221-11.2012 PMC3480225

[31] Tarsa L, Goda Y (2002) Synaptophysin regulates activity-dependent synapse formation in cultured hippocampal neurons. Proc Natl Acad Sci U S A 99: 1012–1016. 11792847 10.1073/pnas.022575999 PMC117422

[32] Kinouchi R, Takeda M, Yang L, Wilhelmsson U, Lundkvist A, et al (2003) Robust neural integration from retinal transplants in mice deficient in GFAP and vimentin. Nat Neurosci 6: 863–868. 12845328 10.1038/nn1088

[33] Lu YB, Iandiev I, Hollborn M, Korber N, Ulbricht E, et al (2011) Reactive glial cells: increased stiffness correlates with increased intermediate filament expression. Faseb J 25: 624–631. 20974670 10.1096/fj.10-163790

[34] Nakazawa T, Takeda M, Lewis GP, Cho KS, Jiao J, et al (2007) Attenuated glial reactions and photoreceptor degeneration after retinal detachment in mice deficient in glial fibrillary acidic protein and vimentin. Invest Ophthalmol Vis Sci 48: 2760–2768. 17525210 10.1167/iovs.06-1398 PMC2613948

[35] Berg A, Zelano J, Stephan A, Thams S, Barres BA, et al (2012) Reduced removal of synaptic terminals from axotomized spinal motoneurons in the absence of complement C3. Exp Neurol 237: 8–17. 22721768 10.1016/j.expneurol.2012.06.008

[36] Kim DS, Li KW, Boroujerdi A, Peter Yu Y, Zhou CY, et al (2012) Thrombospondin-4 contributes to spinal sensitization and neuropathic pain states. J Neurosci 32: 8977–8987. 22745497 10.1523/JNEUROSCI.6494-11.2012 PMC3408211

[37] Li S, Overman JJ, Katsman D, Kozlov SV, Donnelly CJ, et al (2010) An age-related sprouting transcriptome provides molecular control of axonal sprouting after stroke. Nat Neurosci 13: 1496–1504. 21057507 10.1038/nn.2674 PMC3059556

